# Carotid and Aortic Stiffness in Patients with Heterozygous Familial Hypercholesterolemia

**DOI:** 10.1371/journal.pone.0158964

**Published:** 2016-07-19

**Authors:** Alexandra I. Ershova, Alexey N. Meshkov, Tatyana A. Rozhkova, Maria V. Kalinina, Alexander D. Deev, Anatoliy N. Rogoza, Tatyana V. Balakhonova, Sergey A. Boytsov

**Affiliations:** 1 Department of clinical cardiology and molecular genetics, National Research Center for Preventive Medicine, Moscow, Russia; 2 Department of atherosclerosis, Russian Cardiology Research Center, Moscow, Russia; 3 Department of epidemiology of chronic noninfectious diseases, National Research Center for Preventive Medicine, Moscow, Russia; 4 Department of new methods of diagnostics, Russian Cardiology Research Center, Moscow, Russia; Shanghai Institute of Hypertension, CHINA

## Abstract

**Background:**

The role of plasma cholesterol in impairing arterial function and elasticity remains unclear. We evaluated arterial stiffness, measured locally in the common carotid artery by high-resolution echo-tracking, and aortic stiffness, using carotid-femoral pulse wave velocity (PWV) (the “gold-standard” measurement of arterial stiffness), in treatment-naive patients with heterozygous familial hypercholesterolemia (FH).

**Methods:**

The study included 66 patients with FH (10–66 years old) and 57 first-degree relatives without FH (11–61 years old). Carotid-femoral PWV was determined by SphygmoCor (AtCor, Australia). The parameters of carotid stiffness β-index, Peterson elastic modulus and local PWV were assessed with regard to the common carotid artery at a distance of 1cm from the bifurcation (AlokaProsound Alpha7, Japan).

**Results:**

FH patients showed significantly higher β-index (6.3(4.8–8.2) vs. 5.2(4.2–6.4), p = 0.005), Ep (78(53–111) kPa vs. 62(48–79) kPa, p = 0.006), local PWV (5.4(4.5–6.4) m/c vs. 4.7(4.2–5.4) m/c, p = 0.005), but comparable values of carotid-femoral PWV (6.76(7.0–7.92) m/c vs. 6.48(6.16–7.12) m/c, p = 0.138). Carotid arteries and the aorta stiffened with age in patients with FH, but after 30 years, carotid arteries stiffened more significantly than the aorta.

**Conclusions:**

Our study demonstrated that treatment-naive patients with FH had stiffer carotid arteries than their relatives, but showed no difference in aortic stiffness. We also found out that the rate of reduction of elasticity of the aorta and carotid arteries in FH patients varies: it is observed earlier in carotid arteries than in the aorta.

## Introduction

Hypercholesterolemia is a well-established risk factor for the development of cardiovascular diseases and related complications [1], but the role of plasma cholesterol in impairing arterial function and elasticity remains unclear [2–5]. It is estimated that cholesterol and oxidized low-density lipoprotein cholesterol have a number of direct non-atheromatous effects on the arterial wall, which may lead to arterial stiffening. They increase oxidative stress and inflammation, and promote elastin damage and deposition of calcium within the arterial wall [6–9].

Patients with familial hypercholesterolemia (FH), who have been exposed to a high concentration of plasma cholesterol since childhood, are characterized by premature atherosclerosis [10]. There are controversial data about arterial elasticity in patients with familial hypercholesterolemia [11–13].There are several possible reasons for the controversial data: firstly, studies include patients both with and without previous statin treatment; secondly, healthy control subjects are usually genetically distant from patients with FH; and thirdly, different methods, which differ in accuracy and reproducibility, are used to assess arterial stiffness.

Because it directly reflects arterial stiffness, and has the best predictive value for cardiovascular outcome, carotid-femoral pulse wave velocity (PWV) is now considered the gold standard for assessment of arterial stiffness in daily practice[14]. Only one study has assessed carotid-femoral PWV in patients with FH, and this demonstrated significantly higher values of PWV in patients with FH than in healthy subjects [15].

Echo-tracking systems provide optimal conditions for the precise determination of local arterial stiffness, which is directly determined, requiring no assumption from models of the circulation [16]. There is only one study assessing common carotid artery stiffness by echo-tracking in patients with FH, and this shows that local arterial stiffness is increased in asymptomatic normotensive children with FH, suggesting that hypercholesterolemia plays a key role in arterial mechanical impairment from childhood [17].

We evaluated both arterial stiffness, measured locally in the common carotid artery by high-resolution echo-tracking, and aortic stiffness, measured by carotid-femoral PWV, in treatment-naive patients with heterozygous FH and their FH-free first-degree relatives.

## Materials and Methods

### Subjects

A total of 66 FH patients (10–66 years old) without previous statin treatment (39 probands and 27 of their relatives with FH) and 57 of their first-degree FH-free relatives (11–61 years old) without previous treatment with statins or other hypolipidemic drugs were enrolled in the study. The study included only probands with ‘definite’ and ‘probable’ FH diagnoses, according to the Dutch Lipid Clinic Network criteria, based on clinical findings, personal and familial clinical history, and biochemical variables [18]. A diagnosis of FH in relatives was confirmed if the diagnosis was ‘likely’, according to the diagnostic FH criteria for relatives outlined by the National Institute for Health and Clinical Excellence, based on age, sex and low-density lipoprotein cholesterol level [19]. A diagnosis of FH in relatives was ruled out if the diagnosis was ‘uncertain’ according to the diagnostic FH criteria for relatives outlined by the National Institute for Health and Clinical Excellence. Probands were detected by targeted screening in primary care guided by severe hypercholesterolaemia. Relatives were identified after cascade screening of family members of known index cases.

None of the patients had diabetes mellitus, metabolic syndrome, hypothyroidism, nephritic syndrome, systemic disease, oncological disease, Cushing’s syndrome, anorexia nervosa, or therapy with immunosuppressant drugs or corticosteroids.

In all subjects, anthropometric parameters (height and weight) were measured and body mass index was calculated. The presence of tendon xanthomas and smoking status (smoker or non-smoker) were assessed in all study participants.

Blood pressure was measured on the left arm after the subject had been lying for 10 minutes in the supine position. Blood pressure was measured automatically using an oscillometric device, the Omron M3 Expert (Omron Healthcare Co. Ltd., Japan), validated for use in children and adolescents. The mean value of two assessments was used. Hypertension was defined for a mean systolic blood pressure of at least 140 mmHg, a mean diastolic pressure of at least 90 mmHg, or use of antihypertensive medication.

Coronary artery disease was verified by medical records and physical examination, and, if necessary, a stress test was carried out. Coronary artery disease was confirmed on the basis of the most recent international guidelines [20].

The study was approved by the Ethics Committee of the National Research Center for Preventive Medicine and the Russian Cardiology Research Center. Written informed consent was obtained from the participants; if they were children, consent was also obtained from their parents.

### Laboratory tests

Venous blood sample were drawn from subjects after they had fasted overnight. Total cholesterol, triglycerides, high-density lipoprotein cholesterol, glucose, high-sensitivity C-reactive protein and lipoprotein (a) levels were measured using an automatic biochemistry analyzer, ARCHITECT c8000 (Abbott Laboratories, Abbott Park, IL, USA). Low-density lipoprotein cholesterol was calculated according to the Friedewald formula [21]. Homocysteine levels were determined using an AxSYM immunoassay analyzer (Abbott Laboratories, Abbott Park, IL, USA). Fibrinogen levels were measured using an ACL ELife Pro coagulation analyzer (IL, Milan, Italy).

### Measurement of arterial stiffness

Arterial stiffness was measured according to the recommendations for standardization of subject conditions [16]:cigarette smokers were allowed to participate if they had abstained for at least 3 h; room temperature was 22 ± 1°C; subjects rested for at least 10 min in a recumbent position, and so on. Blood pressure measurement was performed before each new determination of arterial stiffness. The obtained values of systolic and diastolic blood pressure were added to the system to calculate stiffness parameters automatically.

Carotid stiffness β-index, Peterson elastic modulus, arterial compliance, and local carotid PWV were automatically assessed in the common carotid artery at a distance of 1 cm from the bifurcation using echo-tracking software (Aloka Prosound Alpha7, Hitachi-Aloka, Tokyo, Japan) and a 14 MHz linear-type probe. All examinations were performed by the same sonographer. A 45° wedge pillow was used to help standardize lateral rotation of the head.

The sonographer positioned two tracking gates on a two-dimensional ultrasound image of the common carotid artery, at the boundary between the media and adventitia of the far and near walls of the vessel. Three consecutive measurements were taken of the anterior longitudinal view of the right artery and then of the left artery. Between measurements the sonographer removed the probe from the patient’s neck. All acquisitions were synchronized with the electrocardiographic signal. Stroke changes in the artery’s diameter were recorded at 10–15 s. Pulse waves of 12 cardiac cycles were used for analysis, and the data were averaged. Poor-quality pulse waves were excluded from the analysis. The averages of the six values obtained by measurements of both carotid arteries were used for analysis.

Carotid-femoral PWV was measured by the same operator using an established technique and a SphygmoCor system (AtCormedical, West Ryde, NSW, Australia), which, owing to its applanation tonometer, allows both proximal (carotid artery) and distal (femoral artery) pulse waves to be obtained.

### Measurement of IMT and plaque parameters

High-resolution B-mode ultrasonography was performed with a 17-5MHz linear-type probe (PHILIPS iU22 ultrasound system, Philips Inc., Eindhoven, the Netherlands). All measurements were taken in the common carotid artery, carotid bulb and proximal segment of internal carotid artery. Three different longitudinal views (anterioroblique, lateral, and posterior oblique) of both carotid systems and transverse views of all plaques were obtained. A more detailed procedure was described in an earlier publication [22].The individual value of mean-IMT was the mean of mean-IMTs of the right and left carotid arteries.The presence of atherosclerotic plaque was estimated at 6 sites of carotid pool: the whole length of both CCAs, both bifurcations, and both ICAs. Plaque number was defined as the sum total of the plaques. An individual value of maximum percent stenosis was defined as a maximum reduction in the percent diameter stenosis of both carotid arteries.

### Reproducibility

Intraobserver repeatability at short time intervals (the variability of the first and second measurements obtained from the same artery) was evaluated with regard to the measurements obtained from all subjects participating in the study. Coefficients of variation of β-index, Peterson elastic modulus, and arterial compliance values, evaluated on the right and left carotid arteries, varied from 12.7% to 14.6%. Coefficients of variation of local carotid PWV values were 6.6% and 6.9%, respectively, for the right and left carotid arteries. The intraclass correlation coefficient, calculated by SPSS (version 19.0), was very high for all carotid stiffness parameters and local carotid PWV. The smallest value of intraclass correlation coefficient was found for arterial compliance (0.94; 95% confidence interval (CI), 0.92–0.96). Intraobserver differences were also plotted using the method described by Bland and Altman [23].

Reproducibility of carotid-femoral PWV waspreviously described in detail [24].

### Statistical analysis

Univariable statistical analysis was performed with Statistica 6.0 software. A *p-*value of less than 0.05 was considered to be statistically significant. Data are presented as median (25th–75th percentile).

The *p-*value for quantitative parameters was calculated using a non-parametric Mann–Whitney test. The *p-*value for quality parameters was calculated using Yates’ corrected *χ*^2^ test. If the sample size included five subjects or fewer, a two-tailed Fisher’s exact test was used. Correlation was tested using Spearman’s test.

Multiple logistic regression analysis (forward stepwise method) was performed to identify significant risk factors for artery stiffening (SAS software, version 6.12). Upper quartiles of all continuous covariates were used for analysis. The exception was arterial compliance when lower quartile was put to use. The odds ratio (OR) [95% confidence interval (CI)] was calculated by multivariate logistic regression analysis.

## Results

Relatives without FH were matched to patients with FH with respect to age, sex, smoking, systolic and diastolic blood pressure, levels of high-density lipoprotein cholesterol, glucose, high-sensitivity C-reactive protein, fibrinogen, lipoprotein (a), and homocysteine, and the prevalence of hypertension. However, the patients were not matched for body mass index or level of triglycerides. Clinical and laboratory characteristics of the patients are shown in Table 1.

**Table 1:** Characteristics of the subjects.

	FH patients(n = 66)	FH-free relatives(n = 57)	*p-*value
**Age, years**	38 (27–48)	33 (23–42)	0.054
**Men, n(%)**	26(39.4)	26(45.6)	0.608
**Tendonxanthomas, n (%)**	31(47)	0	<0.001
**Body mass index, kg/m**^**2**^	24.3 (21.5–28.2)	22.5(20.4–25.4)	0.020
**Smoking, n(%)**	14(21.2)	10(17.5)	0.777
**Total cholesterol, mmol/l**	9.21 (7.73–10.4)	4.98(4.39–5,53)	<0.001
**Triglycerides, mmol/l**	1.14 (0.84–1.81)	0.83 (0.59–1.07)	<0.001
**High-density lipoprotein cholesterol, mmol/l**	1.48 (1.31–1.68)	1.40(1.16–1.63)	0.446
**Low-density lipoprotein cholesterol, mmol/l**	7.07 (5.51–8.04)	3.16(2.69–3.54)	<0.001
**Glucose, mmol/l**	4.87 (4.56–5.34)	4.81 (4.59–5.22)	0.710
**High-sensitivity C-reactive protein, mg/dl**	0.11 (0.04–0.33)	0.07(0.03–0.16)	0.073
**Fibrinogen, g/l**	3.10 (2.80–3.61)	3.0(2.55–3.3)	0.065
**Lipoprotein(a), mg/dl**	13.60(5.10–36.80)	8.20(4.05–13.05)	0.082
**Homocysteine, μmol/l**	11.08 (9.86–14.42)	10.43(8.75–11.9)	0.113
**Systolic blood pressure, mmHg**	111 (105–122)	109 (101–121)	0.346
**Diastolic blood pressure, mmHg**	73 (67–83)	69 (63–78)	0.064
**Hypertension, n (%)**	14(21.2)	6(10.5)	0.175
**Hypotensive therapy, n (%)**	14(21.2)	6(10.5)	0.175
**Coronary artery disease, n (%)**	6(7.6)	0	0.041

Patients with FH demonstrated significantly higher β-index, Peterson elastic modulus, and local carotid PWV, and significantly lower arterial compliance, but comparable carotid-femoral PWV (Table 2).

**Table 2:** Parameters of carotid and aortic stiffness.

Parameters	FH patients	FH-free relatives	*p-*value
**β-index**	6.3 (4.8–8.2)	5.2 (4.2–6.4)	0.005
**Peterson elastic modulus, kPa**	78(53–111)	62 (48–79)	0.006
**Arterial compliance, mm**^**2**^**/kPa**	0.99 (0.69–1.28)	1.26 (1.0–1.5)	0.002
**Local carotid PWV, m/s**	5.4 (4.5–6.4)	4.7 (4.2–5.4)	0.005
**Carotid-femoral PWV, m/s**	6.76 (6.0–7.92)	6.48 (6.16–7.12)	0.138

Carotid-femoral PWV did not differ significantly between patients with FH and their relatives in various age ranges (Table 3). Whereas β-index, Peterson elastic modulus, and local carotid PWV were significantly higher, arterial compliance was significantly lower in the 30–49-year age group. Differences between patients with FH and their relatives for β-index and arterial compliance were close to significant in the 50–69-year age group.

**Table 3:** Stiffness parameters in various age ranges.

Age ranges	FH patients^a^	FH-free relatives^b^	*p-*value(FH-patients vs. relatives)	*p-*value(different age ranges in FH patients)
**β-index**				
**10–29**	4.3 (3.7–5.2)	4.2 (3.4–4.9)	0.379	-
**30–49**	6.9 (5.9–8.2)	5.9 (5.0–6.9)	0.037	<0.001^c^
**50–69**	9.6 (7.6–11.3)	6.7 (6.6–8.4)	0.061	0.001^d^
**Peterson elastic modulus, kPa**				
**10–29**	51 (44–57)	46 (39–53)	0.294	-
**30–49**	82 (68–111)	70 (60–83)	0.027	<0.001
**50–69**	118 (100–135)	102 (86–114)	0.275	0.001
**Arterial compliance, mm**^**2**^**/kPa**				
**10–29**	1.41 (1.22–1.7)	1.44 (1.28–1.73)	0.667	-
**30–49**	0.93 (0.67–1.05)	1.04 (0.86–1.31)	0.009	<0.001
**50–69**	0.68 (0.6–0.8)	0.97 (0.83–1.01)	0.074	0.010
**Local carotid PWV, m/s**				
**10–29**	4.2 (4.0–4.6)	4.1 (3.6–4.5)	0.406	-
**30–49**	5.6 (5.2–6.4)	5.1 (4.7–5.6)	0.015	<0.001
**50–69**	6.8 (6.3–7.3)	6.1 (5.9–6.6)	0.150	0.001
**Carotid-femoral PWV, m/s**				
**10–29**	6.0 (5.2–6.7)	6.2 (5.2–6.5)	0.928	-
**30–49**	7.3 (6.4–8.1)	6.9 (6.5–7.3)	0.275	0.001
**50–69**	7.6 (6.7–8.5)	7.5 (6.7–8.0)	0.861	0.270

^a^FH patients: 24 individuals aged 10–29 years, 27—aged 30–49 years, 15—aged 50–69 years.

^b^Control group: 25 individuals aged 10–29 years, 27—aged 30–49 years, 5—aged 50–69 years.

^c^10-29 vs. 30–49 years

^d^30-49 vs. 50–69 years

Parameters of carotid and aortic stiffness increased significantly with age (Table 4).

**Table 4:** IMT and plaque parameters.

Subjects	FH patients(n = 66)	FH-free relatives(n = 57)	P-value
Mean-IMT, mm	0.55 (0.50–0.70)	0.51 (0.46–0.64)	0.028
Plaque number	2 (0–5)	0 (0–1)	0.000
Maximum percent stenosis, %	38 (0–48)	0 (0–27)	0.000

**Fig 1:**
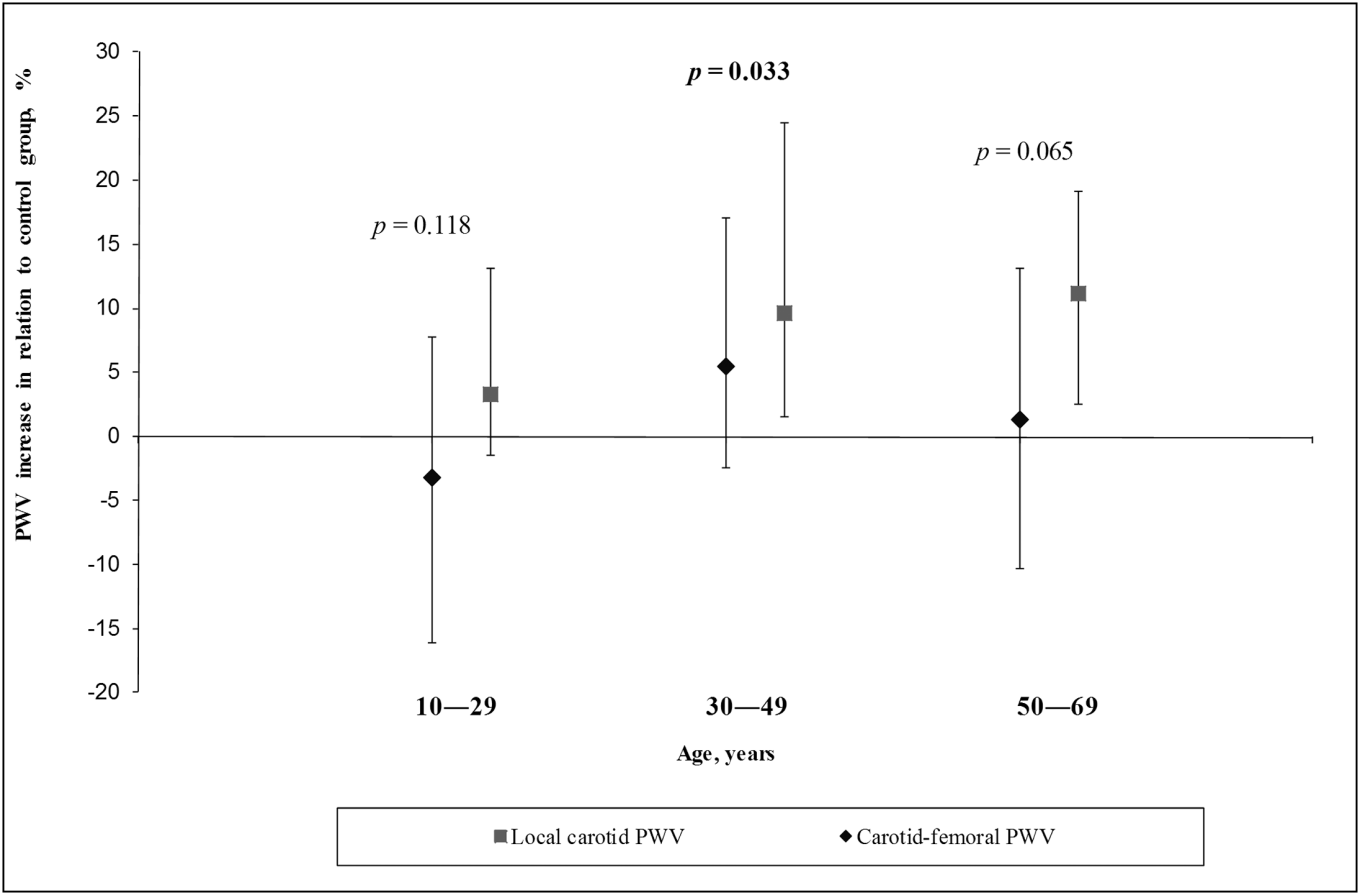
Increase of carotid and aortic PWV in relation to the control group.

We calculated the ratio between each FH patient’s values of local carotid PWV and carotid-femoral PWV and values representing the 50th percentile of the spread of values in the control group within the relevant age ranges. We identified that local carotid PWV increased more significantly than carotid-femoral PWV in patients with FH who were older than 30 years (Fig 1).

The correlation of local carotid PWV and carotid-femoral PWV was moderate (*r* = 0.53, *p*< 0.001).

**Fig 2:**
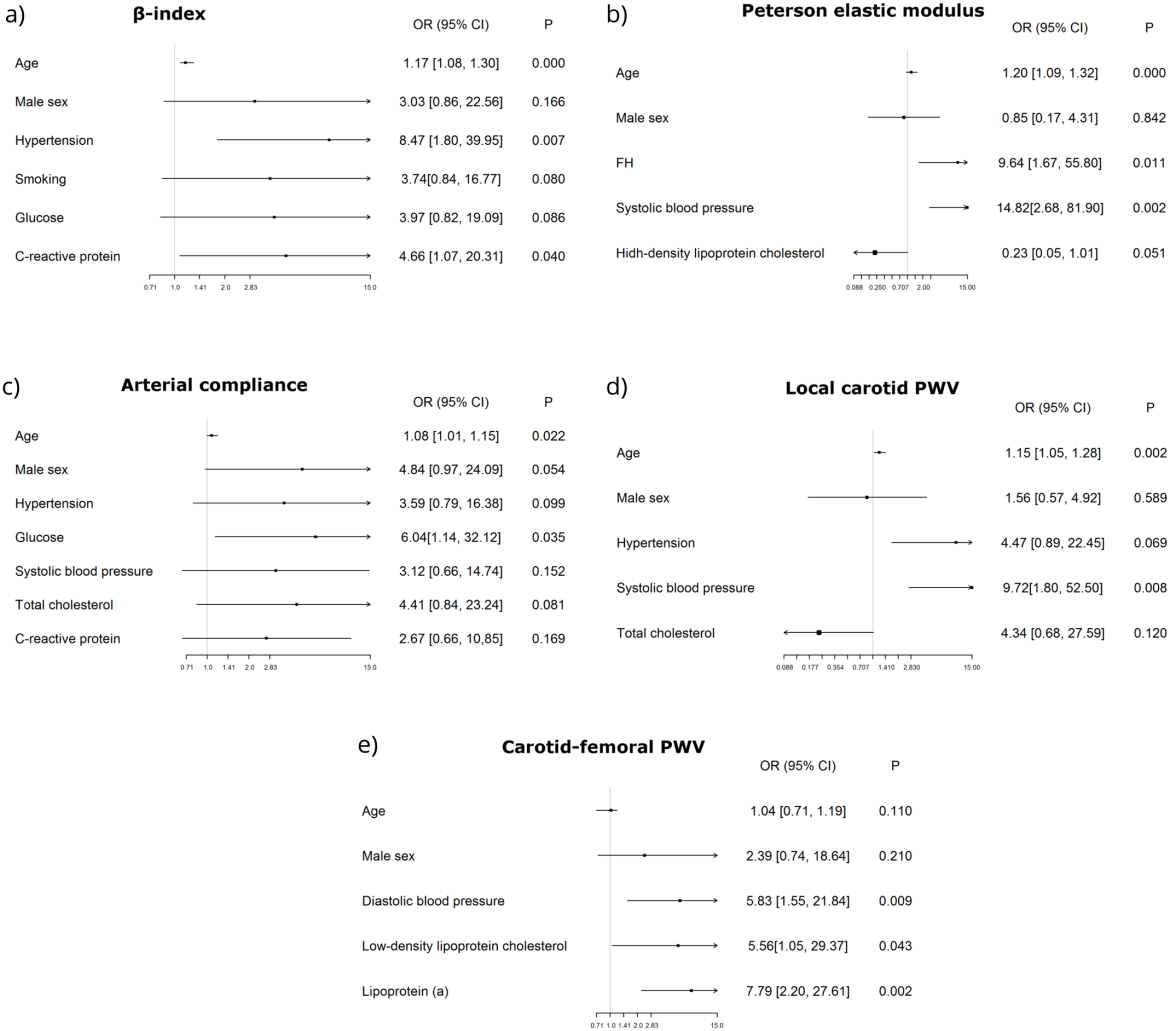
Factors associated with increased arterial stiffness: multiple logistic regression analysis.

Multiple logistic regression analysis demonstrated that stiffness parameters associated not only with basic and well-known factors such as age and arterial pressure, but also with FH, low-density lipoprotein cholesterol, C-reactive protein, and glucose (Fig 2).

IMT, plaque number, and maximum percent stenosis were significantly higher in patients with FH in compared to FH-free relatives (Table 4).

## Discussion

In this study we have shown that the arterial stiffness parameters, measured locally in the common carotid artery using high-resolution echo-tracking, were higher in treatment-naive patients with heterozygous FH than in their FH-free first-degree relatives. Aortic stiffness, measured with carotid-femoral pulse wave velocity, was comparable in patients with FH and their FH-free relatives.

Similar data regarding local stiffness assessment by echo-tracking in patients with FH were obtained by Riggio et al [17]. However, unlike our study, they analyzed only the group of children, whereas the control group included only healthy subjects.

In the only study reported on carotid-femoral PWV in patients with FH, it was shown that carotid-femoral PWV was significantly higher in patients with FH (89 men, aged 39 ± 14 years) than in the control group (31 men, aged 40 ± 12 years), but, in contrast to our study, 31.5% of patients received statin therapy [15]. Perhaps the differences between study groups were obtained because the control group in this study included more healthy subjects than the number of FH-free relatives included in our study.

In the only study assessing the stiffness of both the aorta and the carotid arteries in patients with FH, no significant differences were obtained either for aortic or for carotid stiffness [13]. However, the methods of arterial stiffness measurement in that work differed from those used in our study: compliance was measured in the thoracic aorta by magnetic resonance imaging and in the common carotid artery by B-mode ultrasound. Moreover, the results were obtained from a very small sample of patients (10 men).

The differences obtained in our study could be explained by different methodological approaches to the study of different vascular beds. However, in our study, the stiffness was measured by reproducible and validated methods. One of these methods is considered to be the ‘gold-standard’ for measuring arterial stiffness and the other one (echo-tracking) is more reliable than the standard B-mode ultrasound method [16]. Paini et al., using the same methods to estimate local and regional stiffness, showed that stiffness of the aorta increased with age more significantly than stiffness of the carotid artery in patients with hypertension or type 2 diabetes [25]. The fact that, in common with Paini et al., we obtained stiffness discrepancies for different arterial systems refutes arguments against the use of different methodological approaches. Perhaps features of pathophysiologic mechanisms play a key role in stiffening of arteries in patients with FH as well as in patients with hypertension and type 2 diabetes.

It should be noted that the carotid-femoral PWV was higher than the local carotid PWV, which conflicts with pathophysiologic features of arterial wave propagation. It is well-known that the amplitude of the pressure wave is higher in peripheral arteries than in the central arteries [16]. Found discrepancies can be explained by the fact that one value (carotid-femoral PWV) was obtained by indirect measurement, and the other (local carotid PWV) was calculated.

In previous studies, the association between stiffness parameters and age was demonstrated [26], but the variation of stiffness in different age groups was not analyzed. We also identified that carotid and aortic stiffness in patients with FH accelerates with age. Significant differences between carotid stiffness in patients with FH and their FH-free relatives were found in the 30–49-year age group, and in the 50–69-year age group the differences were close to significant. The absence of differences in the older age group may be attributed on the one hand to the significance of genetic factors decreasing with age, and on the other hand to the small sample sizes of comparison groups in this age range.

In our study, it was shown that the rate of change in carotid and aortic stiffness is variable: after 30 years, carotid stiffness increases faster than aortic stiffness. The results indicate that in patients with FH the carotid arteries are involved in the process of elasticity reduction to a greater extent than the aorta. Moreover, considerable changes in arterial stiffness occur after childhood, when severe hypercholesterolemia has already been observed. The aorta is an elastic vessel, whereas the carotid arteries are musculoelastic. As a result of its morphological characteristics, the aorta is exposed to a greater variability in stiffness than the carotid arteries, whereas the carotid arteries are more exposed to the atherosclerotic process. We suppose that atherosclerosis is the primary process in patients with FH, and that the increase in arterial stiffness is secondary to atherosclerosis. Cholesterol and oxidized low-density lipoprotein cholesterol probably have direct effects on the vascular wall in patients with FH, but this effect is insignificant.

There is data that the magnitude of the effect of each risk factor varies among the arterial beds: for example, hypertension selectively augments atherosclerosis of the cerebral arteries [27], whereas smoking selectively augments atherosclerosis of the abdominal aorta and the iliac and femoral arteries [28]. Patients with FH are specific patients. Besides the fact that they are exposed to the same risk factors as people in the general population, since childhood they have had an important risk factor such as a severe hypercholesterolemia. Therefore, it can be assumed that the development of atherosclerosis in these patients can have its particularities. For instance, in the study of Odink AE et al, hypercholesterolemia was a significant risk factor for coronary and carotid calcification and was not a factor for aortic one in women[29]. Soljanlahti S et al compared aortic elasticity and plaques in carotid and femoral arteries between 19 FH patients, 18 type 2 DM patients, and 30 controls, all aged 48 to 64[30]. They concluded that compliance of the descending aorta was the lowest in DM patients and highest in controls, with no significant differences between FH and DM patients. Carotid and femoral IMT was greater in FH and DM patients than in controls. FH patients had more plaques and were the only ones with stenoses in carotid arteries. They theorized that the differences between FH and DM reflect the differences between atherosclerosis and arteriosclerosis.

Consequently, pathogenetic mechanisms of atherosclerosis development have an effect on the differences in carotid and aortic stiffness. A moderate (not strong) correlation between local carotid PWV and carotid-femoral PWV is an additional argument proving that the factors affecting carotid and aortic stiffness differ.

Therefore, we have suggested that measurement of local carotid stiffness is better than assessment of aortic PWV for the estimation of arterial stiffness in FH patients.

Our study has several limitations. Body mass index was significantly higher in the group of patients than in the relatives. Hence, stiffness parameters were not associated with body mass index by multiple regression analysis. On the one hand, taking into account previously identified associations between artery stiffness and obesity [31], these intergroup differences in the level of body mass index are important. On the other hand, most of the patients had normal weight or were overweight but not obese. In addition, small sample sizes were required, to obtain full information on the age peculiarities of stiffness in FH patients.

At first glance, the disadvantage of this study could be its design. Our investigation is a cross-sectional study. It may appear that the best design for analysis of changes in arterial stiffness with age is a prospective study design. However, our study cannot be a prospective one. The distinctive feature and the advantage of our work is that we studied FH patients without previous statin therapy. We evaluated the natural changes of vessel wall elasticity as a result of prolonged severe hypercholesterolemia. FH is associated with increased risk of cardiovascular events, so as soon as the diagnosis of FH has been established, all FH patients were put on statins. It is known that statins slow the progression of atherosclerosis and have beneficial effects on arterial stiffness [32–33].

Advantages of our study also involve the inclusion of FH-free relatives as a comparison group, and the use of the most reliable methods for arterial stiffness assessment.

## Conclusions

Patients with FH are characterized by persisting hypercholesterolemia. Information regarding the impact of hypercholesterolemia on arterial elasticity is contradictory. In our study, we examined elasticity of the aorta and carotid arteries. An increase in arterial stiffness is typical for patients with FH. As a result of morphological differences in the vascular walls, it is possible that the aorta and carotid arteries respond differently to such a triggering pathogenic factor as durably persisting severe hypercholesterolemia. After 30 years of age the stiffness of the carotid arteries increases faster than aortic stiffness.To estimate arterial stiffness in patients with FH, we suggest assessment of local carotid stiffness is of greater informative value than assessment of aortic PWV.
